# Preparation of Barium-Hexaferrite/Gold Janus Nanoplatelets Using the Pickering Emulsion Method

**DOI:** 10.3390/nano11112797

**Published:** 2021-10-22

**Authors:** Jelena Papan, Patricija Hribar Boštjančič, Alenka Mertelj, Darja Lisjak

**Affiliations:** 1 Department of Complex Matter, Jožef Stefan Institute, Jamova Cesta 39, 1000 Ljubljana, Slovenia; Patricija.Hribar.Bostjancic@ijs.si (P.H.B.); alenka.mertelj@ijs.si (A.M.); darja.lisjak@ijs.si (D.L.); 2 Vinča Institute of Nuclear Sciences, National Institute of the Republic of Serbia, University of Belgrade, P.O. Box 522, 11001 Belgrade, Serbia; 3 Jožef Stefan International Postgraduate School, Jamova Cesta 39, 1000 Ljubljana, Slovenia

**Keywords:** barium hexaferrite, magnetic nanoplatelets, Granick’s method, Janus nanoparticles, gold

## Abstract

Janus particles, which have two surfaces exhibiting different properties, are promising candidates for various applications. For example, magneto-optic Janus particles could be used for in-vivo cancer imaging, drug delivery, and photothermal therapy. The preparation of such materials on a relatively large scale is challenging, especially if the Janus structure consists of a hard magnetic material like barium hexaferrite nanoplatelets. The focus of this study was to adopt the known Pickering emulsion, i.e., Granick’s method, for the preparation of barium-hexaferrite/gold Janus nanoplatelets. The wax-in-water Pickering emulsions were stabilized with a combination of cetyltrimethyl ammonium bromide and barium hexaferrite nanoplatelets at 80 °C. Colloidosomes of solidified wax covered with the barium hexaferrite nanoplatelets formed after cooling the Pickering emulsions to room temperature. The formation and microstructure of the colloidosomes were thoroughly studied by optical and scanning electron microscopy. The process was optimized by various processing parameters, such as the composition of the emulsion system and the speed and time of emulsification. The colloidosomes with the highest surface coverage were used to prepare the Janus nanoplatelets by decorating the exposed surfaces of the barium hexaferrite nanoplatelets with gold nanospheres using mercaptan chemistry. Transmission electron microscopy was used to inspect the barium-hexaferrite/gold Janus nanoplatelets that were prepared for the first time.

## 1. Introduction

Since 1991, when Pierre-Gilles de Gennes spoke about them in his Nobel lecture [1], Janus particles have been the object of investigations for many different research groups. What makes Janus particles attractive is their dual nature. The surfaces of these particles have two different sides which can be individually designed to exhibit distinct functionalities in a single particle. Janus particles can be used in catalysis, drug delivery, sensing, nanomachines, anti-bacterial applications, specific cell targeting, chemo-photothermal therapy, etc. [2,3,4,5,6]. Of particular interest are magnetic Janus nanoparticles that combine surface anisotropy with magnetic properties [7]. For example, if magnetic Janus particles were to be coupled with plasmonic particles (gold, silver), they could be used in biomedicine for magnetic targeting and simultaneous optical diagnostics, photothermal therapy, stimuli-responsive drug delivery, surface-enhanced Raman spectroscopy (SERS), controlling bleeding, etc. [8,9,10,11].

According to the literature, there are three main approaches to obtain Janus nanoparticles: masking, phase separation, and self-assembly using various techniques, such as electrohydrodynamic co-jetting systems, surface modification, etc. [12]. One of the simplest methods for the production of Janus particles is the masking method, where one side of the particle is protected and the other is available for further chemical modification. Masking is a reversible process and allows for the collection of Janus particles. There are two types of masking methods. The first one involves a solid substrate and evaporative deposition, electrostatic adsorption, or “polymer single-crystal templating”. The second one is the immobilization of nanoparticles at the interface of two fluid phases, such as the Pickering emulsion method [13]. In a Pickering emulsion, particles accumulate at the interface between two immiscible liquids and stabilize the droplets against coalescence. The supra-colloidal structures obtained in the Pickering emulsion are called colloidosomes [14,15]. One of the widely used Pickering emulsion methods is Granick’s method, which was proposed for a wax-in-water system with silica particles. The emulsion was prepared by mixing two phases at temperatures above the wax’s melting point, during which silica particles adsorbed onto the wax-water interface to form a stable Pickering emulsion. The system was subsequently cooled to room temperature to solidify the emulsion (i.e., wax) droplets, while the silica particles remained fixed at the wax surface. The unmasked sides of the silica particles were subsequently chemically modified [16]. Granick’s method was improved by the use of a cationic surfactant for tuning the hydrophilicity of the particles. At the same time, the surfactant directly influences the penetration depth of the particles into the wax droplets and, thus, the exposed surface area of the particles [17].

Many different kinds of Janus particles have been produced using Granick’s method because it is an inexpensive method for synthesizing Janus particles in relatively large quantities. Examples of the fabrication of Janus particles using Granick’s method are given in Table 1 [18,19,20,21,22,23,24,25]. The focus of most of these studies was on possible applications of the Janus particles [19,20,21,23]. However, the preparation of Pickering emulsions with Granick’s method is not simple. The main problem is that Pickering emulsions are thermodynamically sensitive systems, and many external factors interfere with the process of emulsification and the preparation of colloidosomes. Only a few articles have focused on the processing parameters, for example, [26], where the production of colloidosomes with a monolayer coverage was optimized with spherical silica particles. In particular, the surface coverage of the wax with core particles is very important because it directly influences the production of Janus particles [27]. If we have a monolayer coverage, we will only have Janus particles as the main product, but if we have a multilayer coverage, we will have a mix of Janus particles and unmodified core particles.

Thermodynamically, the efficiency of the particles in stabilizing the Pickering emulsions originates from their spontaneous adsorption at the interphase [28]. The adsorption of the solid particles at the oil-water interface requires partial wetting of the solid by the water and the oil; this is a matter of the interfacial energies of the three interfaces: solid-water, solid-oil, and oil-water. If the particles are hydrophilic, they will be in the water phase, but if they are hydrophobic, they will prefer the oil phase. Tuning the hydrophilicity of the nanoparticles (e.g., with a surfactant) is necessary to stabilize the Pickering emulsions. The contact angle θ between the particle and interface depends on its wetting properties θ < 90° for hydrophilic particles, θ > 90° for hydrophobic particles) [29]. The free energy of adsorption ∆Gads is related to the contact angle, the tension between the two phases (γ), and the size (d—diameter) of the particles. The planar area of oil-water interface can be reshaped by the presence of the particle. For a small enough particle (i.e., nanoparticle) with negligible gravity, the oil-water interface remains planar up to the contact line with the particle. Therefore, the free energy of adsorption, ∆Gads, is given in Equations (1) and (2) (for spherical particles) [30]:

∆G_ads_ (sphere) = −(π/4) d^2^ γ(1 − |cos θ|)2 for θ < 90° (1)

∆G_ads_ (sphere) = −(π/4) d^2^ γ(1 + |cos θ|)2 for θ > 90° (2)

The adsorption of particles at the oil-water interface is the strongest when the contact angle is 90° [30]. According to Equations (1) and (2), the adsorption free energy is always larger than the thermal energy (kT = 4.11 × 10^−21^ J at 293 K), and this means that the particle adsorption is a spontaneous process. The free energy of adsorption decreases noticeably with a decrease in particle size. Therefore, Pickering emulsions are typically produced with (sub) micron particles [29,31]. In contrast to this, nanoplatelets (NPLs) can stabilize Pickering emulsions [32]. For an NPL of negligible thickness, the free energy of adsorption is given by the following [33]:

∆G_ads_ (platelet) = −(π/4) d^2^ γ(1 − |cos θ|) (3)

where d is the circular diameter of the NPLs. Compared to spheres, the adsorption energy of the platelets is higher when the contact angle is not 90°. This means that NPLs are potentially better emulsion stabilizers than the spherical particles. It has previously been proven that NPLs tend to minimize their surface area and energy by lying flat at the interface [33].

The adsorption rate of particles at the oil-water interface is also an important parameter for the stabilization of Pickering emulsions. Although the adsorption of particles at a fluid interface is thermodynamically favored, the process can be too slow in real experiments. If the adsorption rate is slower than the coalescence rate of the droplets, then the droplets coalesce before being stabilized [34]. A slower adsorption rate indicates a high energy barrier against adsorption [35]. In real oil-water systems, the adsorption energy barrier is usually so high that Pickering emulsions can only be made by applying vigorous mechanical stirring [28]. The use of high-dispersion processors significantly increases the adsorption rate because it enormously increases the number of interactions between the particles and the interface at a given time, and thus, the probability that the particles overcome the adsorption-energy barrier significantly increases.

Only a few kinds of Janus NPLs have been made, where the NPLs were clay (kaolinite, laponite), zirconium hydrogen phosphate, milled silica, polymer, or silver, but such Janus NPLs were not prepared using the Pickering emulsion method [32,36,37,38,39,40]. Magnetic Janus particles were prepared only from spherical, superparamagentic core nanoparticles (e.g., Fe_3_O_4_) [21,23,24]. However, there are no published studies (to the best of our knowledge) of Janus NPLs made with a hard magnetic core.

Our goal was to optimize Granick’s method for the production of Janus NPLs where the hard magnetic barium hexaferrite NPLs are decorated with gold nanospheres on only one of the two basal planes. Gold nanospheres were selected as a proof-of-concept since they allow for the direct observation of the Janus character of the studied NPLs with transmission electron microscopy.

## 2. Materials and Methods

### 2.1. Materials

Barium (II) nitrate (99.95%), scandium (III) nitrate hydrate (99.9%), iron (III) nitrate nonahydrate (98+%), citric acid (99+%), tetraethylorthosilicate (99%), sodium hydroxide (98%), and hydrogen tetrachloroaurate (III) trihydrate (99+%) were purchased from Alfa Aesar, Lancashire, UK. Paraffin wax (mp ≥ 65 °C) was purchased from Aldrich, St. Louis, MO, USA, cetyltrimethylammonium bromide CTAB (>99%) was obtained from VWR Int. GmbH, Vienna, Austria, chloroform was purchased from Merck KGaA, Darmstadt, Germany, and 3-mercaptopropyltriethoxysilane (95%) was purchased from Gelest, Morrisville, PA, USA. Nitric acid (65%), ethanol (99.5%), sodium hydroxide, and ammonium hydroxide (25%) were purchased from Carlo Erba Reagents S.A.S, Milan, Italy. All the chemicals were used without any further purification.

### 2.2. Synthesis

Our core particles were silica-coated barium hexaferrite NPLs (NPLs-Si). Barium hexaferrite (BaFe_12_O_19_) with a partial substitution of Fe^3+^ with Sc^3+^ (BHF NPLs) were prepared via a hydrothermal route, colloidally stabilized in water with citric acid, and then coated with silica via Stöber’s method [41,42]. This method is a sol-gel process for the preparation of silica coatings where the silica precusors are silicon alkoxides, such as tetraethyl orthosilicate (TEOS) [43]. After the purification, water dispersions of NPLs were used in the emulsification process.

To prepare the Janus NPLs, Granick’s method was used, with modifications applied to a few of the procedures [16,17,18,19,20]. More specifically, the CTAB aqueous solution was slowly added to the aqueous NPLs-Si dispersion under mechanical stirring [26]. The CTAB stock aqueous solution was used with a concentration of 0.192 mM, i.e., far below the critical micelle concentration (CMC) [44]. The NPLs-Si dispersion was then heated to 80 °C, at which point the molten wax was added (previously melted in an oven at 100 °C) and the emulsification process was enabled with a high shear disperser (T25 digital ULTRA-TURRAX with the dispersion tool S 25 EC-T-C-18G-ST and an integrated temperature sensor, Staufen, Germany) [26]. The temperature of the system was kept constant. After the emulsification, the mixture was cooled and the resulting colloidosomes were filtered, washed several times with water and ethanol, and then dried. Wax colloidosomes (Samples 1–9) were prepared with the conditions listed in Table 2.

The unmasked side of the NPLs-Si was coated with (3-mercaptopropyl) trimethoxysilane (MPTMS) as follows: the wax colloidosomes (1 g) from the previous step were dispersed in an ethanol/water mixture (2:1), the pH was adjusted to be between 8 and 9, and then an ethanolic solution of MPTMS was added [19,45]. The mixture was stirred overnight at room temperature. After mixing, the wax colloidosomes were washed with water and ethanol and dispersed in 20 mL of water. Next, 200 µL of 17 nm citrate-capped Au nanoparticles was added to this dispersion, prepared using the classic citrate method [46]. The mixture was stirred for 3 h, then filtered, and subsequently washed with water and ethanol. In the next step, the wax was dissolved with chloroform. The Janus NPLs were extracted with water, centrifuged at 35,000 rcf/10 min with a supercentrifuge (Thermo Scientific Sorvall LYNX 6000 Superspeed Centrifuge, Leicestershire, UK), and magnetically separated from the unbonded Au nanospheres. Finally, the Janus NPLs were dispersed in water and stored in a glass vial.

### 2.3. Characterization

The NPLs were analyzed with a transmission electron microscope (TEM, Jeol 2100, with EDXS spectrometer JED 2300 EDS- Akishima, Tokyo, Japan). Their widths were estimated from the TEM images with a visual measurement using DigitalMicrograph™ Gatan Inc. Plaesanton, CA, and expressed as an equivalent diameter. Electro-kinetic measurements (zeta-potential) of the NPLs dispersed in deionized water were monitored using a ZetaPALS instrument (Brookhaven Instruments Corporation, Holtsville, NY, USA). Wax colloidosomes were analyzed with an optical microscope (Olympus BX51M, Tokyo, Japan) and a scanning electron microscope (SEM, Jeol 7600, Akishima, Tokyo, Japan). Dynamic light scattering (DLS) analysis of the NPLs in water was performed with an Anton Paar Litesizer 500, Graz, Austria. The magnetic properties of the dried NPLs were measured with a vibrating sample magnetometer (VSM, Lakeshore 7407, Westerville, OH, USA). The dried NPLs-Si and NPLs-Si coated with mercaptan were analyzed using Fourier transform infrared (FTIR) spectroscopy with a PerkinElmer Spectrum 400 FTIR/FT-FIR spectrometer, Buckinghamshire, United Kingdom. The spectra of the dried samples were taken with a universal attenuated total reflectance (ATR) sampling accessory in the range between 4000 and 650 cm^−1^. The UV-Vis spectra of the Janus NPLs in water were measured with a HP 8453 UV-Vis spectrometer, Poway, CA, USA.

## 3. Results and Discussion

### 3.1. Optimization of the Emulsification Process

The as-synthesized BHF NPLs used for the preparation of the Janus NPLs had an average size of 47 ± 21 nm (Figures S1–S3). They are a hard magnetic material, with a room temperature saturated mass magnetization of 35 A m^2^ kg^−1^ and a coercivity of 97 kAm^−1^ (Figure S4). Their magnetoplumbite crystal structure was confirmed via X-ray diffractogram; this structure was similar to those obtained in our previous studies (for example, in [41]). In order to colloidally stabilize the NPLs, we coated them with citric acid and silica to be used in all the subsequent experiments. The thickness of the silica layer was approximately 2 nm (Figure S2).

Our NPLs-Si were hydrophilic and negatively charged (Figure S5), so they were not directly suitable for making Pickering emulsions [26,47]. To promote the adsorption of NPLs-Si onto the wax-water interface, we had to tune their hydrophilicity with surfactants. It has been experimentally proven that air-water and oil-water interfaces are negatively charged. Therefore, depending on the ionic strength, negative particles adsorb onto such interfaces either very slowly or not at all because they are repelled by them, whereas positively charged particles adsorb readily [28,48]. Hydrophilicity and the negative charge of the particles can be decreased with a cationic surfactant, and the most commonly used cationic surfactants are cetyltrimethylammonium bromide (CTAB) and dimethyldidodecylammonium bromide (DDAB) [17,23,49]. The hydrophilic-lipophilic balance (HLB) index of CTAB is 10; for the DDAB surfactant, it is 18.1. According to the HLB index, CTAB is a better option [50,51] because the most suitable surfactants for an oil-in-water (O/W) emulsion should have an HLB index of between 8 and 18 [52]. Therefore, in our study, we used CTAB to control the adsorption of the NPLs-Si at the wax-water interface.

**Figure 1:**
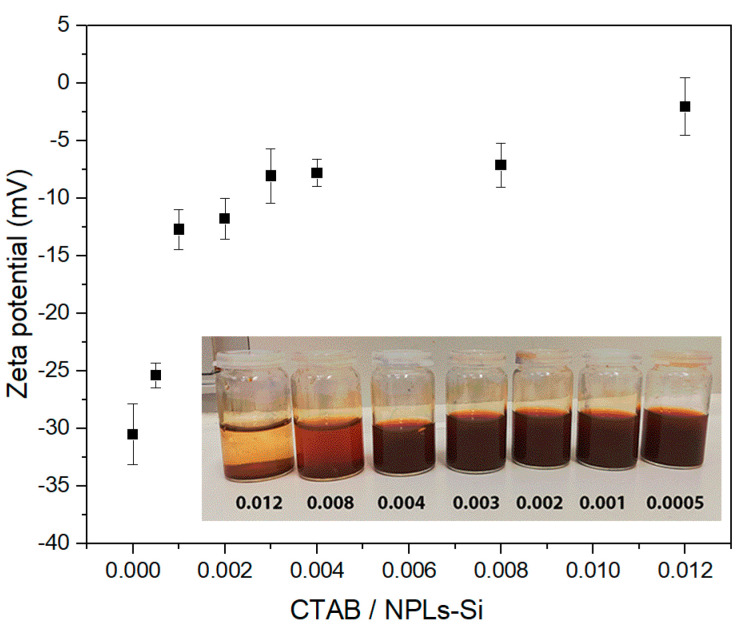
Zeta-potential vs. CTAB/NPLs-Si mass ratio. The inset shows suspensions with different ratios of CTAB/NPLs-Si, as noted at the bottom of the photograph.

The influence of the CTAB concentration on the zeta-potential of NPLs-Si is given in Figure 1. CTAB dramatically changed the zeta-potential of the NPLs-Si suspensions. The largest increase of the zeta-potential (from −30.5 ± 2.7 to −25.4 ± 1.1 mV) was observed for the smallest CTAB addition (i.e., CTAB/NPLs-Si ratio = 0.0005; Figure 1). The CTAB/NPLs-Si ratio is defined as CTAB/NPLs-Si ratio = mass of CTAB/mass of NPLs-Si. The surfactant CTAB interacts with the NPLs-Si through an electrostatic interaction between the positively charged surfactant headgroups and the negatively charged siloxane groups. First, small concentrations of CTAB adsorb onto the surface of the NPLs-Si as a monolayer via electrostatic interactions. At larger concentrations of CTAB, more CTAB adsorbs onto the surface of the NPLs-Si (CTAB/NPLs-Si ratio = 0.003) and the zeta-potential slowly increases towards 0 mV. Commonly, particles with a lower surface charge (i.e., less negative) tend to be more suitable for the stabilization of Pickering emulsions. However, a zeta-potential that is too low can also induce aggregation of NPLs on droplets after the emulsion is formed [53]. The aggregation is a consequence of high CTAB concentrations, where the CTAB could adsorb onto the silica surface as a double layer as a result of hydrophobic interactions along the carbon chains. In our experiments, visible aggregation of NPLs was observed for CTAB/NPLs-Si ratio = 0.004 (zeta-potential = −7.8 ± 1.1 mV). Based on the above results, the optimal CTAB/NPLs-Si ratio range is between 0.0005 and 0.004, for which NPLs-Si are expected to have the optimum interaction with the wax droplets, without any visible aggregation.

After tuning the hydrophilicity of the NPLs-Si, we investigated how different parameters (fractions of NPLs, surfactant, water, and wax in the emulsion mixture; stirring speed; and time of treatment) influence the emulsion’s stability and the coverage of the wax droplets by the NPLs-Si. We prepared various sets of wax-in-water emulsions where only one processing parameter was varied in each set. In our first set of experiments, we varied the fractions of NPLs-Si (Table 2: Samples 1–3).

**Figure 2:**
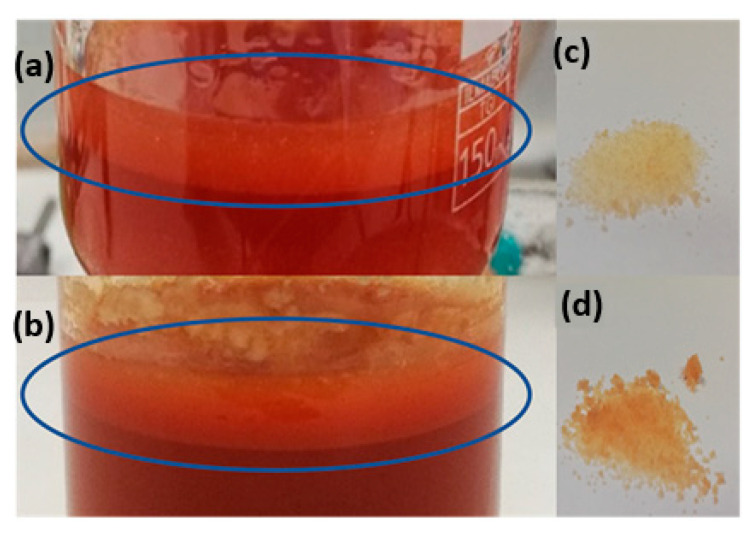
Examples of emulsions—(**a**) Sample 2 and (**b**) Sample 3—and washed wax colloidosomes of (**c**) Sample 2 and (**d**) Sample 3. The emulsion phase is circled in panels (**a**,**b**).

In all three cases, we obtained emulsions (top layer (circled) in Figure 2a,b), and a large fraction of NPLs-Si remained in the water phase (bottom layer in Figure 2a,b). However, after cooling and washing the colloidosomes from the emulsions, only a small fraction of NPLs-Si remained attached at the colloidosomes (see the pale color of the washed colloidosomes in Figure 2c,d in comparison with the intense color of the emulsions in Figure 2a,b). Colored solidified wax colloidosomes were the main evidence that we prepared an oil-in-water emulsion mixture (Figure 2c,d). This color difference was especially significant in the emulsions with a larger fraction of NPLs-Si. The wax colloidosomes of Sample 1 were completely white, while the colloidosomes of Samples 2 and 3 were yellow and pale orange, respectively. Surprisingly, the largest fraction of NPLs-Si remained attached to the colloidosomes in Sample 3, which had the lowest concertation of NPLs-Si. This can be correlated with the different concentrations of surfactant (9 × 10^−5^%, 4 × 10^−5^%, and 3 × 10^−5^ wt.% for Samples 1, 2, and 3, respectively), since the ratio of CTAB/NPLs-Si for all three samples was kept the same (CTAB/NPLs-Si = 0.001). Therefore, Sample 3 was used for the subsequent optimization.

The ultraturax homogenizer induces a high mechanical shear, which helps to overcome the adsorption energy barrier to form a Pickering emulsion [34]. However, a high mechanical shear also results in a high polydispersity of the emulsion droplets, which can lead to the coalescence of the droplets and decreased stability of the emulsions [54]. To prevent the coalescence of the colloidosomes, we had to improve the surface coverage of the colloidosomes with NPLs-Si by optimizing the emulsion droplet size with the efficient adsorption of the NPLs-Si at the interface. This was done by varying the stirring time, surfactant fraction, stirring speed, and wax to water ratio.

**Figure 3:**
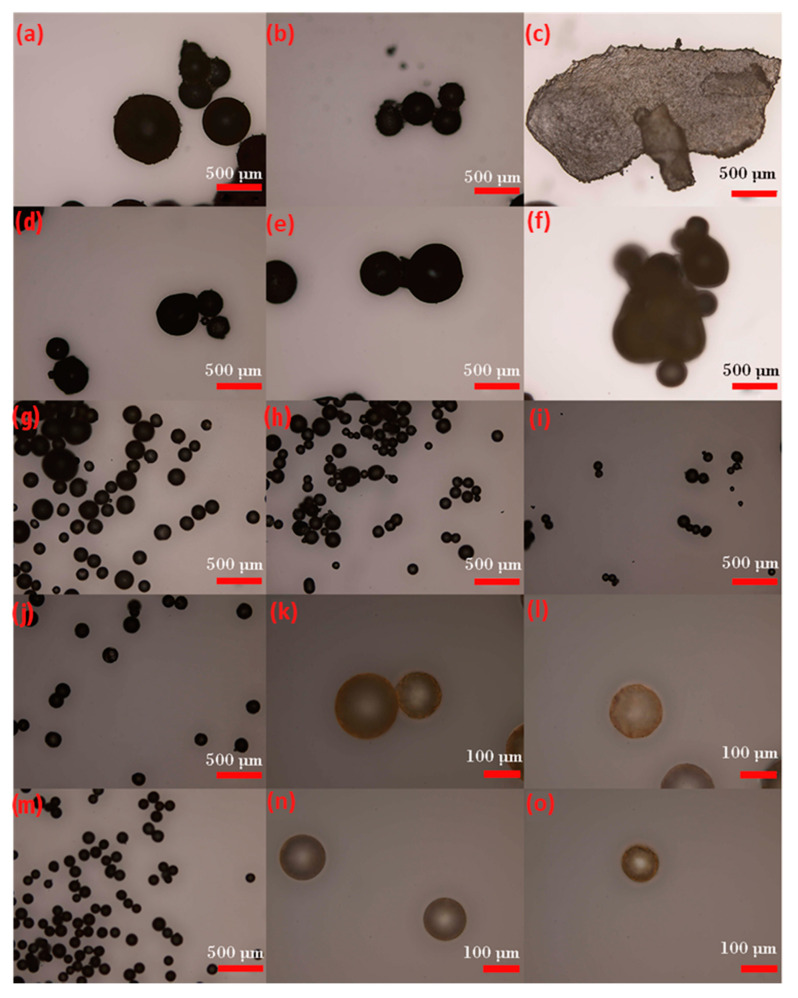
Optical images of Sample 3 (**a**–**c**), Sample 4 (**d**–**f**), Sample 5 (**g**), Sample 6 (**h**), Sample 7 (**i**), Sample 8 (**j**–**l**), and Sample 9 (**m**–**o**).

The optimum speed and time of mixing are two of the most important parameters in the formation of stable and well-covered colloidosomes. According to Table 1, this should be possible at a high stirring speed during a short time of emulsification. At first, we applied stirring at 9000 rpm for 6 min, and the temperature of initial system was 79 °C, but the wax had already solidified during the emulsification. This was because of the uncontrolled cooling of the system, i.e., the temperature at the end of emulsification was below 65 °C. The cooling problem was overcome by a speed reduction from 9000 rpm after the first 3 min to 6000 rpm for another 3 min (Sample 3). The temperature during the whole process was in the range of 70–80 °C. After the emulsification, as the system cooled down to room temperature, the colloidsomes of Sample 3 solidified. They were relatively large, with diameters between 200 and 600 µm (Figure 3a,b). Aside from the colloidosomes, there were also wax pieces of irregular shape, which are an indication of an unstable emulsion (Figure 3c). For the preparation of Sample 4, we prolonged the emulsification time (35 min) and reduced the stirring speed (3000 rpm). The stirring speed of 3000 rpm allowed us to maintain a constant system temperature (i.e., 75–80 °C) for a longer time, which was not possible at a higher stirring speed. The longer emulsification time resulted in smaller colloidosomes than in Sample 3 (Figure 3d). A similar effect was observed in the emulsions made with graphene oxide sheets, where the sizes of the droplets decreased on average and were more uniform when using a longer emulsification [55]. A small fraction of bridged wax spheres was also observed in Sample 4 (Figure 3e,f), which suggests that some of the colloidosomes were only partially covered by the NPLs-Si. During the collisions of partly covered wax, the colloidosomes can coalesce [56]. This effect could be overcome by increasing the CTAB concentration.

**Figure 4:**
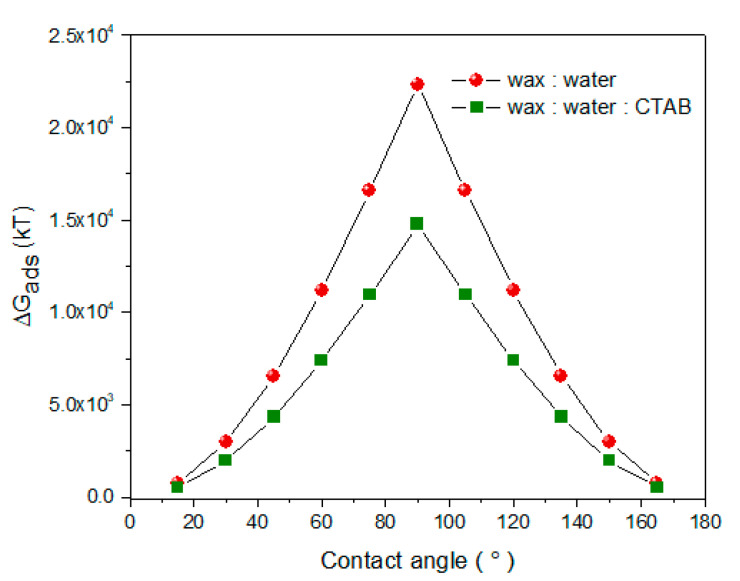
Adsorption energy (∆G_ads_) of BHF NPL at the wax-water interface, (with and without surfactant CTAB) for various contact angles between the wax and water.

The influence of the CTAB concentration on the size of the colloidosomes and the adsorption energy of the NPLs-Si onto the wax droplets was compared in Samples 4–6, while keeping all the other parameters fixed. As can be seen from the images in Figure 3e (Sample 4), Figure 3g (Sample 5), and Figure 3h (Sample 6), an increase in the amount of surfactant led to a decrease in the colloidosomes’ diameters, from 150–550 µm (Sample 4) to 100–500 µm (Sample 5), and then to 90–250 µm (Sample 6). Also, an increase in the CTAB concentration led to a slight decrease (absolute value) of the zeta-potential, from −12.7 ± 1.7 mV (Sample 4) to −11.8 ± 1.8 mV (Sample 5), and then to −8.08 ± 2.3 mV (Sample 6) (corresponding to CTAB/NPLs-Si ratio = 0.001, 0.002, and 0.003, respectively in Figure 1). However, the main impact of the higher CTAB concentration was a decrease in the interfacial tension between the two phases, resulting in a lower energy required for the emulsification [57]. The reported surface tension between wax and water is γ = 53 mN/m, which decreases with the addition of CTAB (with a ratio of CTAB/NPLs-Si = 0.003) to γ = 35 mN/m [58]. For our NPLs-Si, with an average size of 47 ± 21 nm (Figure S3), considering the above surface-tension values, the adsorption energy varies with the contact angles, as presented in Figure 4. As expected, the NPLs-Si are the most strongly held at the interface at the 90° contact angle. The addition of CTAB reduced the energy-adsorption barrier by 34% (Figure 4). Therefore, the optimum ratio of CTAB/NPLs-Si = 0.003 (Sample 6–9) led to a stabile emulsion, without any coalesced colloidosomes. In this case, the adsorption energy of the NPLs-Si must have been lowered just enough to enable the irreversible adsorption of the NPLs-Si at the interface [59].

**Figure 5:**
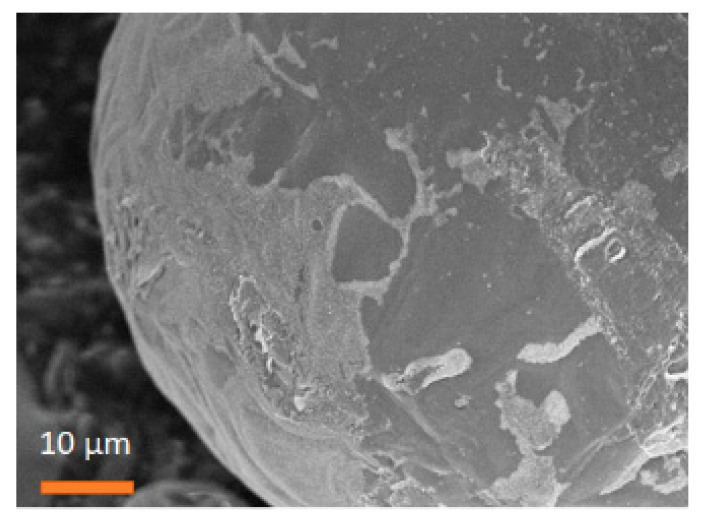
SEM image of Sample 7.

The influence of the stirring speed of the homogenizer on the production of colloidosomes was compared for Samples 6 and 7 (Table 2). It was shown that a higher stirring speed of the homogenizer generates a finer dispersion of the emulsion droplets, with a more uniform size of colloidosomes [26]. In our experiment, the high-speed treatment (3 min at 9000–12,000 rpm) after the regular speed (32 min at 3000 rpm) of emulsification resulted in smaller colloidosomes with diameters in the range of 70–200 µm (Sample 7, Figure 3i). These colloidosomes had a more homogeneous size and shape than the colloidosomes obtained with the lower-speed treatment for the preparation of Sample 6 (Figure 3h). The more homogeneous colloidosomes of Sample 7 were further analyzed with the SEM, which revealed that the colloidosomes were partially covered with NPLs-Si (Figure 5). For the production of the Janus NPLs, colloidosomes covered with a dense monolayer of NPLs-Si are preferred, in order to enable a homogenous functionalization of the non-masked part of the NPLs-Si with the highest possible yield.

**Figure 6:**
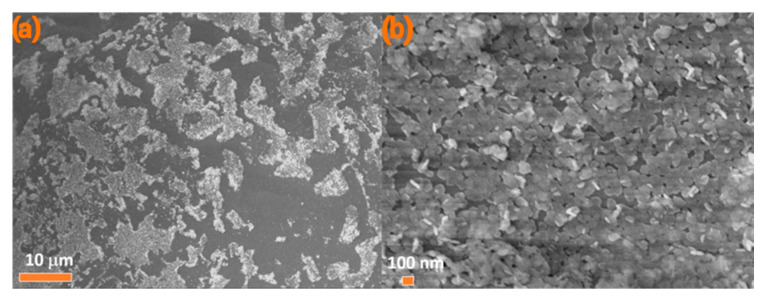
(**a**) SEM image of Sample 8 and (**b**) the magnification of (**a**).

**Figure 7:**
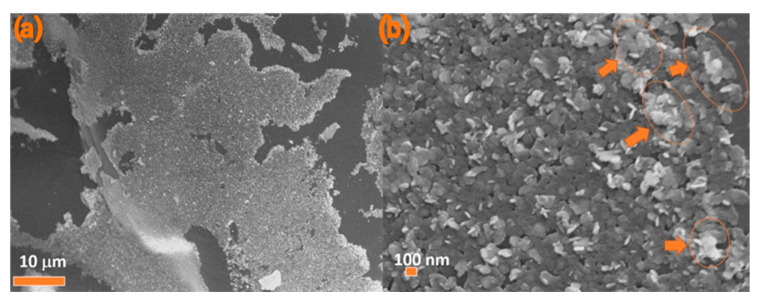
(**a**) SEM image of Sample 9 with (**b**) the magnification of (**a**) with arrows pointing at the aggregates of NPLs-Si in areas circled in orange.

The wax to water ratio (o/w—oil/water) also influences the final emulsion microstructure and determines the interface area between the wax and the water available to be stabilized by the NPLs-Si [49]. The impact of the o-w interface on the colloidosomes’ coverage was compared for Samples 7–9. For all three samples, the diameter of the colloidosomes was similar (70–200 µm) (Figure 3i–o), and the interface area between the o-w interface decreased in the following order: Sample 7 > Sample 8 > Sample 9. The colloidosomes were not homogeneously covered by the NPLs-Si in any of the samples (Figure 5, Figure 6 and Figure 7). The NPLs-Si were assembled in large surface patches of various size at the colloidosomes. We very roughly estimated (with an inspection using the SEM) a larger number of colloidosomes in Samples 7–9, where a higher surface coverage was obtained in Samples 8 and 9 than in Sample 7. From a closer look at the large surface patch covered with NPLs-Si, we can conclude that the NPLs-Si are assembled preferentially in a monolayer. Figure 6b and Figure 7b show examples of such an area, where most of the NPLs-Si lie flat on the wax. The deviation from a monolayer assembly is larger at the borders of the surface patches (Figure 7b) (orange circled areas). So, we selected Samples 8 and 9 for the production of the Janus NPLs. The results are presented in the Section 3.3.

### 3.2. Mechanism of Adsorption of NPLs-Si onto Wax

**Scheme 1:**
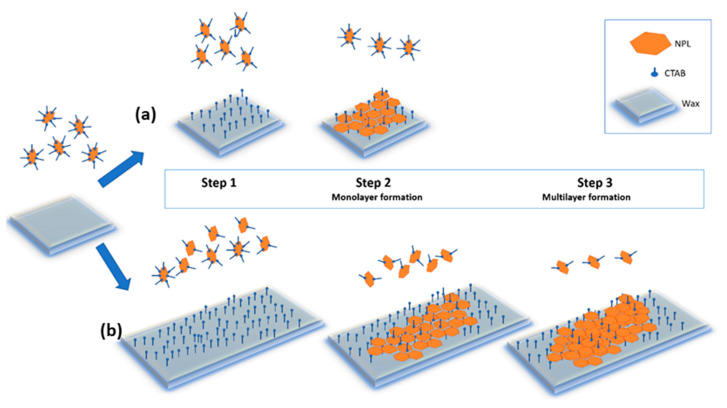
Proposed mechanism of the formation of (**a**) monolayer and (**b**) multilayer NPLs on the wax surface.

The emulsification starts by assembling the CTAB and NPLs-Si onto the o-w interface. The diffusion and, consequently, the assembly of small CTAB molecules are faster than those of the molecules partly desorbed from the NPLs-Si; at the same time, the surface charge of the NPLs-Si becomes more negative (Figure 1). Such NPLs-Si are attracted to the CTAB molecules at the interface and assemble onto the o-w interface (Scheme 1, step 2). On the other hand, the stability of the NPLs-Si in water is increased due to the higher negative surface charge, and some fraction of the NPLs-Si remains dispersed in the water phase.

We propose a mechanism for the adsorption of NPLs-Si and their assembly at the wax surface to explain the different surface coverage for Samples 7–9. Our system has four components: NPLs-Si, CTAB, wax, and water. Furthermore, there is a dynamic equilibrium present between all four phases: CTAB-NPLs-Si↔CTAB-water↔CTAB-wax [26]. The emulsification starts by assembling the CTAB and NPLs-Si onto the o-w (oil-water) interface. The diffusion and, consequently, the assembly of small CTAB molecules are faster than those of the NPLs-Si, and in a very short instant, only the CTAB molecules are adsorbed at the interface (Scheme 1, step 1). At the same time, the concentration of CTAB in the water decreases. Consequently, CTAB partly desorbs from the NPLs-Si to re-establish the CTAB-NPLs-Si↔CTAB-water equilibrium. Because of such desorption, the surface charge of the NPLs-Si becomes more negative (Figure 1). Such NPLs-Si are attracted to the CTAB molecules at the interface and assemble onto the o-w interface (Scheme 1, step 2). On the other hand, the stability of the NPLs-Si in the water increases due to the higher negative surface charge, and some fraction of the NPLs-Si remains dispersed in the water phase. The consequence of the two opposite effects is limited coverage of wax with the NPLs-Si (Figure 5, Figure 6 and Figure 7), with some remaining fraction of the NPLs-Si in the water phase (Figure 3a,b). The described processes are additionally influenced by the size of the o-w interface area (Scheme 1, step 3). The probability of complete coverage of the wax is the smallest for Sample 7, with the largest o-w interface area (i.e., the largest o/w fraction, Table 2). This coincides with our rough estimation from the SEM analyses, where a higher surface coverage of colloidosomes was observed in Samples 8 and 9 than in Sample 7.

Figure 6b and Figure 7b show that the adsorbed NPLs-Si do not assemble into a perfect monolayer. The aggregation of the NPLs-Si onto the firstly adsorbed layer of the NPLs-Si can originate from the magnetic interactions between the adsorbed NPLs-Si and the NPLs-Si in the water phase (Scheme 1b, Step 3). Multilayers of stabilizing particles were also observed in the Pickering emulsions made with kaolinite and laponite platelets and hydrophobic silica particles [34,35,60]. The multilayers formed, most likely, due to the pre-aggregation of the particles in the aqueous phase. However, this was not the case in our study, as confirmed by the DLS measurements. Only a small difference between the average hydrodynamic size and the size distribution of the NPLs-Si was measured with DLS in the water (60 ± 10 nm) and the water-CTAB solution (66 ± 11 nm) (Figure S6). If we consider the average size of the core NPLs obtained from the TEM (47 ± 21 nm), the CTAB, and the solvation layer around the silica-coated NPLs, these results are in reasonable agreement; the CTAB did not induce any significant aggregation.

This aggregation can also occur during the assembly of NPLs at an o-w interface by strong capillary interactions, as suggested by J. C. Loudet et al. [61]. A closer look at the NPLs-Si assembly on colloidosomes (Figure 7b) reveals an almost perfect alignment of the NPLs-Si in the very first layer at the sphere surfaces. Some tilted/aggregated NPLs-Si are present in the subsequent layers. This suggests that the NPLs-Si, primarily remaining in the water phase, must have attached to the already-adsorbed monolayer, most probably via strongly attractive magnetic interactions [62]. Our NPLs-Si exhibited typical hard magnetic behavior (Supplementary Figure S4). We also note that the SEM observation does not necessarily coincide with the situation in the emulsion, as the system conditions change during the processing, i.e., during the cooling of the emulsion, as well as the washing and drying of the colloidosomes. Nevertheless, to produce Janus NPLs, the SEM observation is perfectly relevant, since the surface modification takes place on the colloidosomes, i.e., on the exposed surfaces of the NPLs-Si.

### 3.3. Janus BHF NPLs

**Figure 8:**
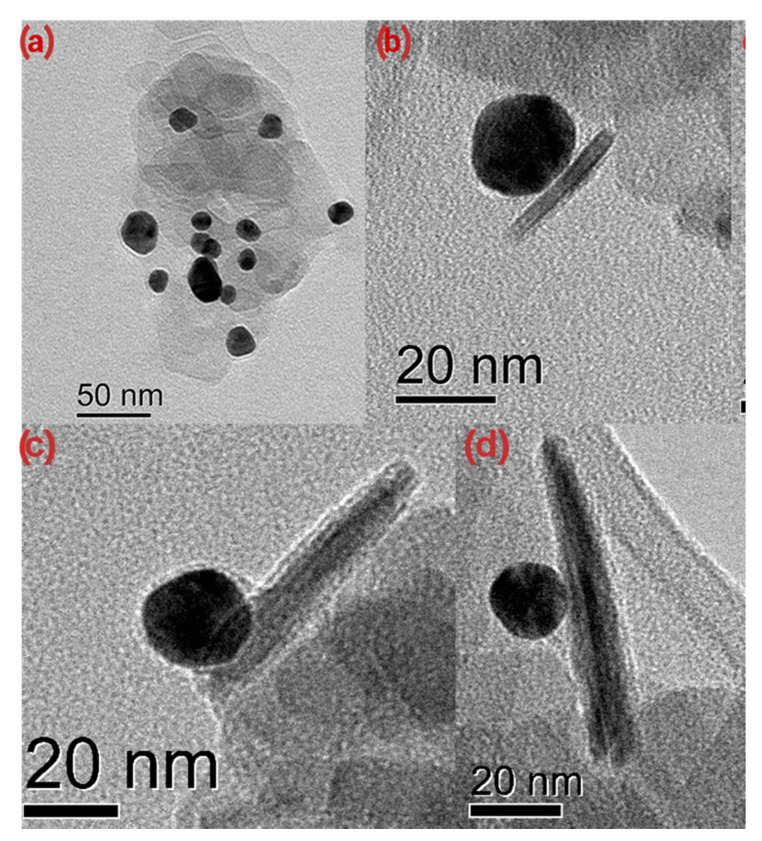
(**a**) Janus NPLs decorated with Au on only one side; (**b**–**d**) a single barium hexaferrite/gold Janus NPL.

The best wax colloidosomes (Sample 8) were used to produce the Janus NPLs. They were first reacted with mercapto-silane to enable linkage with the Au nanospheres [46]. Evidence of the mercapto groups at the surface of the NPLs-Si is shown in the FTIR spectra (Figure S7). The NPLs-Si has a band at 950 cm^−1^ attributed to the Si-OH groups, and when they are coated with mercapto-silane, this band disappears and new bands appear at 1060 cm^−1^ (attributed to the Si-O bond) and 2928 cm^−1^ (related to the C-H stretching deriving from the alkyl chain of MPTMS), and the typically very weak peak related to the S-H group is located at 2600 cm^−1^ [50,63]. Au nanospheres were synthesized with a citrate method (TEM image of Au nanospheres, Figure S8) [46]. It was expected that the surface citrate ligands from Au nanospheres would be exchanged by mercaptanes from the NPLs-Si surfaces, and thus, the two kinds of particles would bond via the mercapto group [64,65]. TEM images of the first Janus barium-hexaferrite/gold particles are given in Figure 8. EDS analysis given in Figure S9 confirms all the constituent elements—Ba, Fe, Sc, Si, and O for the NPLs-Si and Au for the Au nanoparticles. All the Au spheres were attached to the NPLs-Si and no free Au spheres were observed. The presence of Au on only one side of the NPLs-Si is clear evidence of the Janus character of the nanoparticles and a confirmation of the successful optimization of Granick’s method for the production of hard magnetic Janus NPLs.

**Figure 9:**
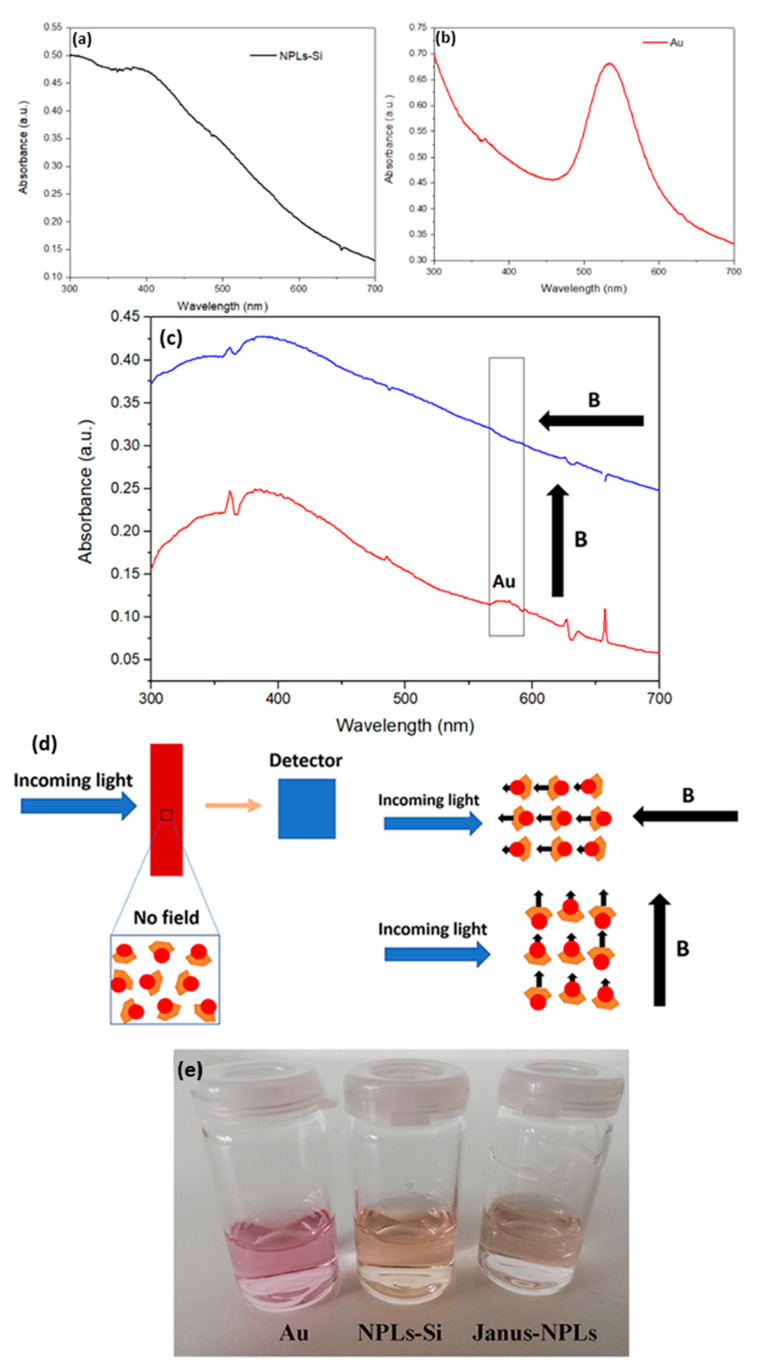
UV-Vis spectra of (**a**) NPLs-Si and (**b**) Au nanospheres; (**c**) UV-vis spectra of Janus NPLs in the presence of parallel (blue line) and perpendicular (red line) magnetic fields; (**d**) Scheme of UV-vis measurement with non-polarized light and applied magnetic field, parallel or perpendicular to the direction of the incident light; (**e**) photograph of aqueous suspensions of Au nanoparticles, NPLs-Si, and Janus NPLs.

Figure 9 shows the UV-vis spectra of the NPLs-Si, gold, and Janus NPL particles prepared in this work and dispersed in water. NPLs-Si (Figure 9a) show a broad absorption below 600 nm, which is the strongest at 300–400 nm and originates from the core BHF NPLs [66]. The Au nanoparticles show typical absorption at around 530 nm due to the surface plasmon resonance (Figure 9b) [67]. The optical properties of the Janus NPLs are demonstrated under an applied magnetic field (Figure 9c), and the scheme of the experiment is given in Figure 9d. We measured the UV-vis absorption with non-polarized light using a magnetic field applied parallel or perpendicular to the direction of the incident light. The external magnetic field aligns the NPLs-Si; therefore, the Janus NPLs are also aligned. When the magnetic field is parallel to the incident light, the NPLs are aligned perpendicular to the incident light and absorb strongly [66], and no distinct absorption by the Au nanoparticles was observed (Figure 9c). On the contrary, in a perpendicular magnetic field, the Janus NPLs are aligned in parallel to the incident light. Consequently, the absorption peak at 576 nm was observed due to the lower absorbance of the NPLs-Si. The original Au absorption peak at 530 nm (Figure 9b) shifted to 576 nm in the Janus NPLs. Such a significant red shift was observed previously in hybrid systems where the magnetic particles (Fe_3_O_4_) were coupled with gold via thiol linking [46]. This effect can also be observed from the slightly darker color of the aqueous suspension of the Janus NPLs than that of the Au nanospheres and the NPLs-Si (Figure 9e).

## 4. Conclusions

The well-known Granick’s method was modified by replacing spherical silica particles with hard magnetic barium hexaferrite nanoplatelets (NPLs-Si). We demonstrated how different parameters (phase composition of the emulsion mixture and the processing conditions) influence the formation of Pickering emulsions. The optimum parameters (0.03% mass NPLs-Si, 8.5% wax, 9 × 10^−5^% CTAB; speed of homogenizer and time of emulsification: (1) 3000 rpm, 32 min; (2) 9000–12,000 rpm, 3 min) were chosen to study the assembly of NPLs-Si at wax droplets. The NPLs-Si were assembled primarily in a monolayer, but they also partially formed multilayers. A monolayer was formed when the optimum concentration of CTAB was used for the stabilization of the wax-water interface. Strong magnetic interactions between the NPLs-Si are the most probable reason for the formation of multilayers. The successful optimization of the Granick method was confirmed by the formation of the first Janus NPLs composed of hard magnetic barium-hexaferrite NPLs and gold nanospheres. Also, we demonstrated the magneto-optic properties of the Janus NPLs by switching the absorbance from gold nanospheres on/off by changing the orientation of an applied magnetic field. In the same way, other types of hard magnetic Janus NPLs could be obtained. For example, gold nanospheres can be replaced by titania nanoparticles or other catalyzers to form recyclable magneto-catalyzers, or magneto-dielectric Janus NPLs could be formed with dielectric nanoparticles or highly polar organics.

## Figures and Tables

**Table 1:** Janus particles produced with Granick’s method and the main processing parameters.

Core Particles	Diameter (nm)	Shape of Particles	Mass of Particles (mg)	Wax to Water Ratio	Speed of Treatment (rpm)	Duration of Treatment	Reference
SiO_2_	70	Sphere	200	1:10	9000	80 s	[18]
SiO_2_-NH_2_	172	Sphere	250	1:5	2200	2 h	[19]
Fe_3_O_4_	50–100	Sphere	100	1:60	22,000	300 s	[20]
Graphene oxide	100–1000	Nanosheets	2000	1:10	12,000	12 min	[21]
Fe_3_O_4_@ SiO_2_	45	Sphere	140	1:50	9500	90 s	[22]
Fe_3_O_4_		Sphere	200	1:6	1650	2 h	[23]
SiO_2_	80	Sphere	140	1:50	9500	90	[24]
TiO_2_	155–1000	Sphere	1–5% ww	1:10	15,000	2 min	[25]

**Table 2:** Investigated conditions for the preparation of Pickering emulsions with NPLs.

Sample	Wax to Water Ratio (*w*/*w*)	CTAB to NPLs-Si Ratio (*w*/*w*)	NPLs-Si (%)	Wax (%) *	CTAB (%) *	Speed Regime	Emulsion
1	1:10	0.001	0.09	9.08	9 × 10^−5^	(1) 9000 rpm, 3 min; (2) 6000 rpm, 3 min	Formed, but all the NPLs are rinsed from wax balls
2	1:10	0.001	0.04	9.08	4 × 10^−5^	(1) 9000 rpm, 3 min; (2) 6000 rpm, 3 min	Formed, but most of the NPLs are rinsed from wax balls
3	1:10	0.001	0.03	9.08	3 × 10^−5^	(1) 9000 rpm, 3 min; (2) 6000 rpm, 3 min	Formed
4	1:10	0.001	0.03	9.08	3 × 10^−5^	3000 rpm, 35 min	Formed
5	1:10	0.002	0.03	9.08	7 × 10^−5^	3000 rpm, 35 min	Formed
6	1:10	0.003	0.03	9.08	10^−4^	3000 rpm, 35 min	Formed
7	1:10	0.003	0.03	9.08	10^−4^	(1) 3000 rpm, 32 min; (2) 9000–12,000 rpm, 3 min	Formed
8	1:10.8	0.003	0.03	8.50	9 × 10^−5^	(1) 3000 rpm, 32 min; (2) 9000–12,000 rpm, 3 min	Formed
9	1:12.7	0.003	0.03	7.30	9 × 10^−5^	(1) 3000 rpm, 32 min; (2) 9000–12,000 rpm, 3 min	Formed

* Fractions are calculated from the total mass of a mixture of four components: water, wax, NPLs-Si, and CTAB.

## Supplementary Materials

The following are available online at https://www.mdpi.com/article/10.3390/nano11112797/s1. Figure S1: TEM image of BHF NPLs, Figure S2: TEM image of NPLs-Si, Figure S3: Particle size distribution of BHF NPLs (N total = 316, Particle size 47 ± 21 nm), Figure S4: Magnetic hysteresis of BHF NPLs, Figure S5: Zeta potential vs. pH of negatively charged NPLs-Si, Figure S6: The number-weighted distributions of the hydrodynamic size of NPLs-Si in water (purple) and NPLs-Si in 10^−4^% CTAB aqueous solution (as used in Sample 7) (pink), Figure S7: IR spectra of NPLS-Si and NPLs-Si coated with mercaptosilane, Figure S8: TEM image of gold nanospheres, Figure S9: (**a**) TEM image of the Janus NPLs and (**b**) EDS spectrum of area showed at (**a**).
