# Supplementary material for: Preparation of Barium-Hexaferrite/Gold Janus Nanoplatelets Using the Pickering Emulsion Method
Source: Nanomaterials (Basel). 2021 Oct 22;11(11):2797. doi: 10.3390/nano11112797 (PMC8621987; doi:10.3390/nano11112797)
Supplement: Supplementary file 1 [file nanomaterials-11-02797-s001.zip › nanomaterials-1398948-supplementary.pdf]

Supplementary

Preparation of barium-hexaferrite/gold Janus nanoplatelets using the Pickering emulsion method

Jelena Papan[a, b,*], Patricija Hribar Boštjančič [a, c], Alenka Mertelj[a], Darja Lisjak[a]

[a] *Jožef Stefan Institute, Jamova cesta 39, Ljubljana, Slovenia*
[b] *Vinča Institute of Nuclear Sciences – National Institute of the Republic of Serbia, University of Belgrade, P.O. Box 522, Belgrade, 11001 Serbia*
[c] *Jožef Stefan International Postgraduate School, Jamova cesta 39, Ljubljana, Slovenia*
**Corresponding author:jelena.papan@ijs.si*

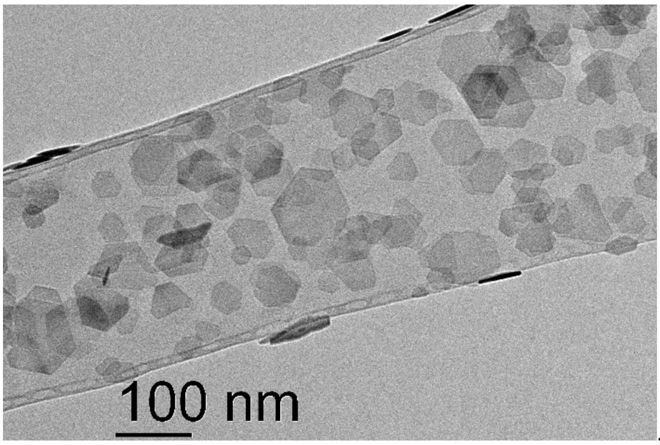

Figure S1. TEM image of BHF NPLs.

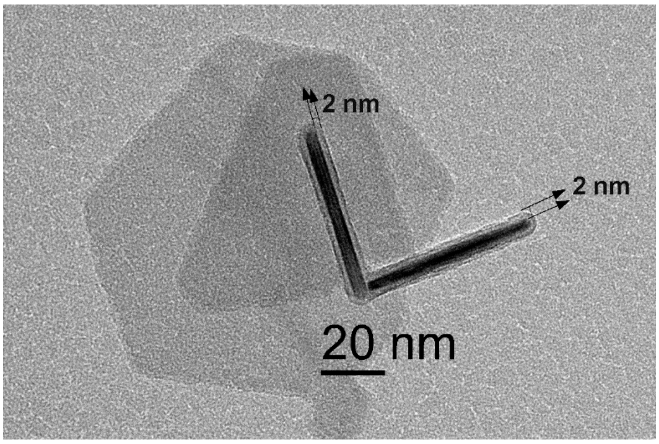

Figure S2. TEM image of NPLs-Si.

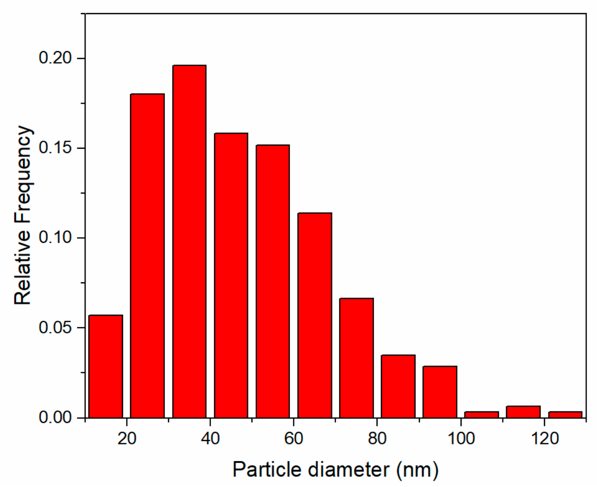

Figure S3. Particle size distribution of BHF NPLs (N total = 316, Particle size 47 ± 21 nm).

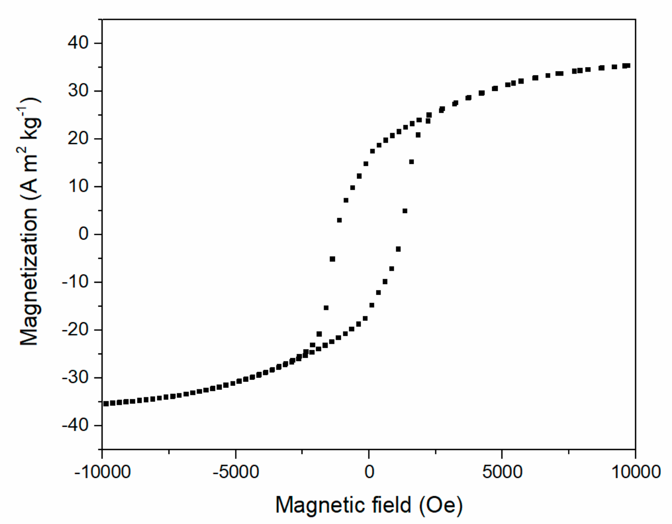

Figure S4. Magnetic hysteresis of BHF NPLs.

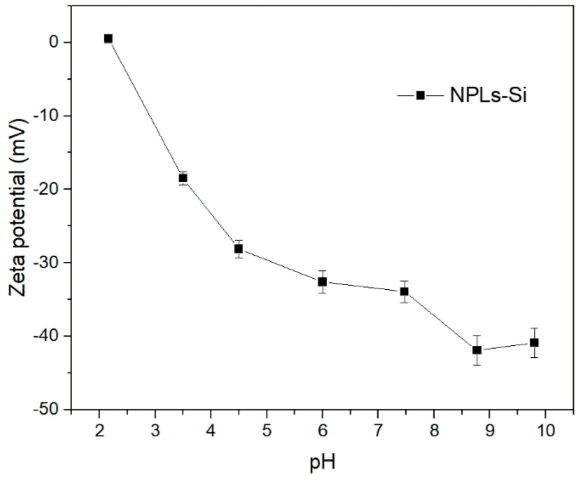

Figure S5. Zeta potential vs. pH of negatively charged NPLs-Si.

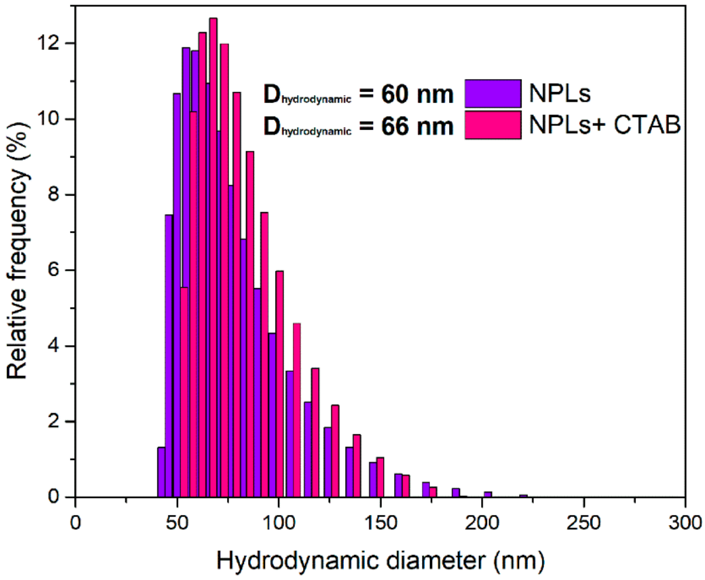

Figure S6. The number-weighted distributions of the hydrodynamic size of NPLs-Si in water (purple) and NPLs-Si in 10^{-4} % CTAB aqueous solution (as used in Sample 7) (pink).

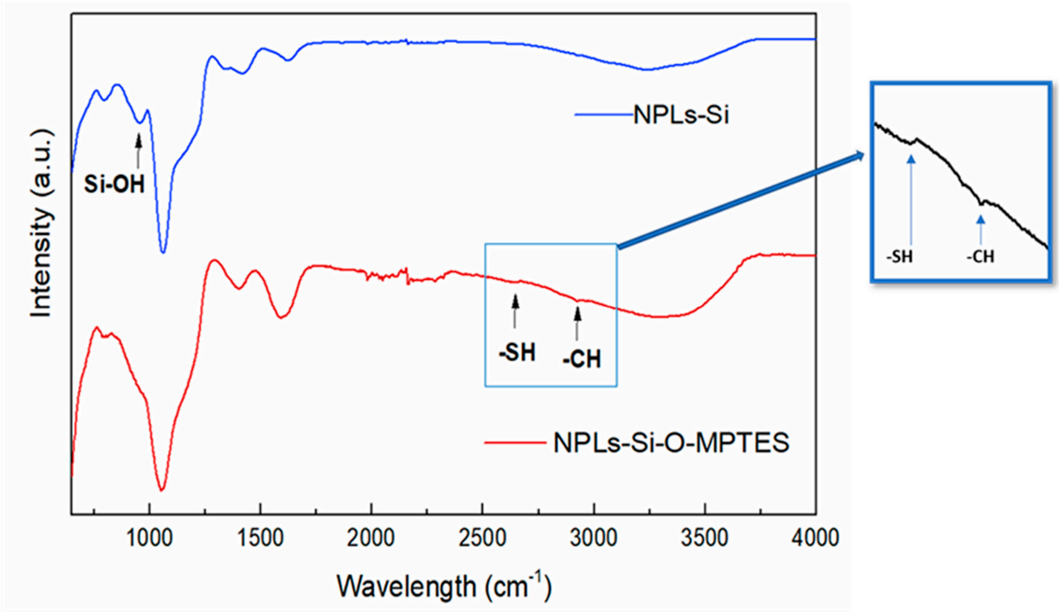

Figure S7. IR spectra of NPLS-Si and NPLs-Si coated with mercaptosilane.

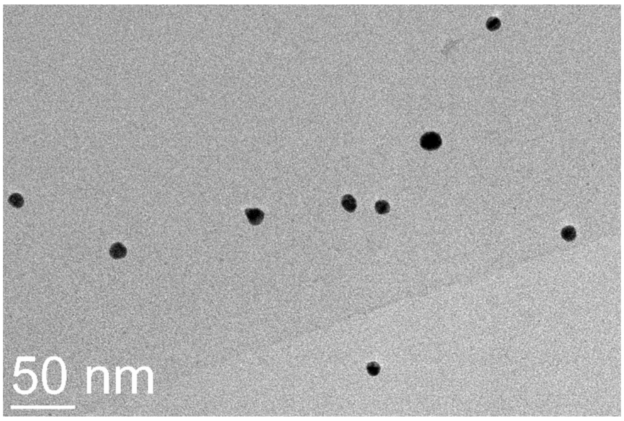

Figure S8. TEM image of gold nanospheres.

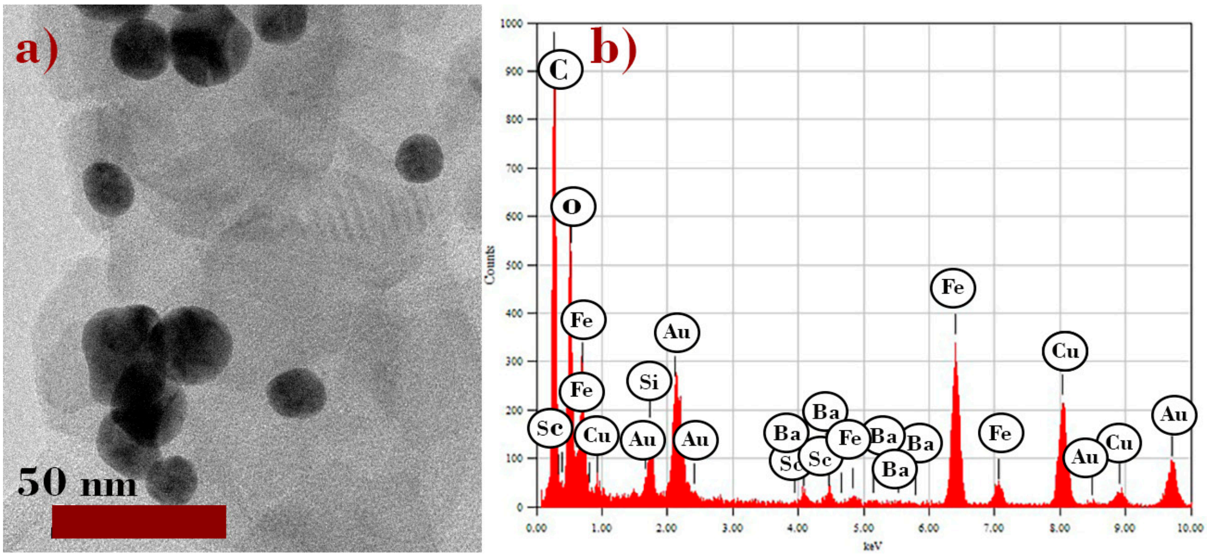

Figure S9. (a) TEM image of the Janus NPLs and (b) EDS analysis of area shown in (a). Cu and C in the EDXS spectrum originate from the sample support grid.
